# Adolescent Alcohol Exposure: Burden of Epigenetic Reprogramming, Synaptic Remodeling, and Adult Psychopathology

**DOI:** 10.3389/fnins.2016.00222

**Published:** 2016-05-31

**Authors:** Evan J. Kyzar, Christina Floreani, Tara L. Teppen, Subhash C. Pandey

**Affiliations:** ^1^Department of Psychiatry, Center for Alcohol Research in Epigenetics, University of Illinois at ChicagoChicago, IL, USA; ^2^Jesse Brown Veterans Affairs Medical CenterChicago, IL, USA; ^3^Anatomy and Cell Biology, University of Illinois at ChicagoChicago, IL, USA

**Keywords:** adolescence, binge drinking, anxiety, epigenetics, neuroinflammation, neurogenesis, dendritic spines

## Abstract

Adolescence represents a crucial phase of synaptic maturation characterized by molecular changes in the developing brain that shape normal behavioral patterns. Epigenetic mechanisms play an important role in these neuromaturation processes. Perturbations of normal epigenetic programming during adolescence by ethanol can disrupt these molecular events, leading to synaptic remodeling and abnormal adult behaviors. Repeated exposure to binge levels of alcohol increases the risk for alcohol use disorder (AUD) and comorbid psychopathology including anxiety in adulthood. Recent studies in the field clearly suggest that adolescent alcohol exposure causes widespread and persistent changes in epigenetic, neurotrophic, and neuroimmune pathways in the brain. These changes are manifested by altered synaptic remodeling and neurogenesis in key brain regions leading to adult psychopathology such as anxiety and alcoholism. This review details the molecular mechanisms underlying adolescent alcohol exposure-induced changes in synaptic plasticity and the development of alcohol addiction-related phenotypes in adulthood.

## Introduction: Adolescent alcohol use and alcohol use disorders

Alcohol exposure, whether acute or chronic in nature, produces profound morphological, structural, and molecular changes in the brain (Spiga et al., 2014a; Kyzar and Pandey, 2015). In the clinic, a pathological cycle occurs with repeated alcohol exposure that is manifested as persistent alcohol use in a chronically relapsing pattern despite related negative consequences (Koob, 2003; Hyman, 2005). The clinical manifestations of alcohol use disorders (AUD) appear to be fueled by neuroadaptations on the molecular level that affect synaptic plasticity on the cellular level and alter connectivity in specific neurocircuitry (Kyzar and Pandey, 2015). A growing understanding of AUD points to the importance of targeting the neurobiological mechanisms underlying synaptic plasticity in the study of AUD pathogenesis, as well as in the development of novel treatment options (Nixon and McClain, 2010; Koob et al., 2014; Kyzar and Pandey, 2015).

**Figure 1:**
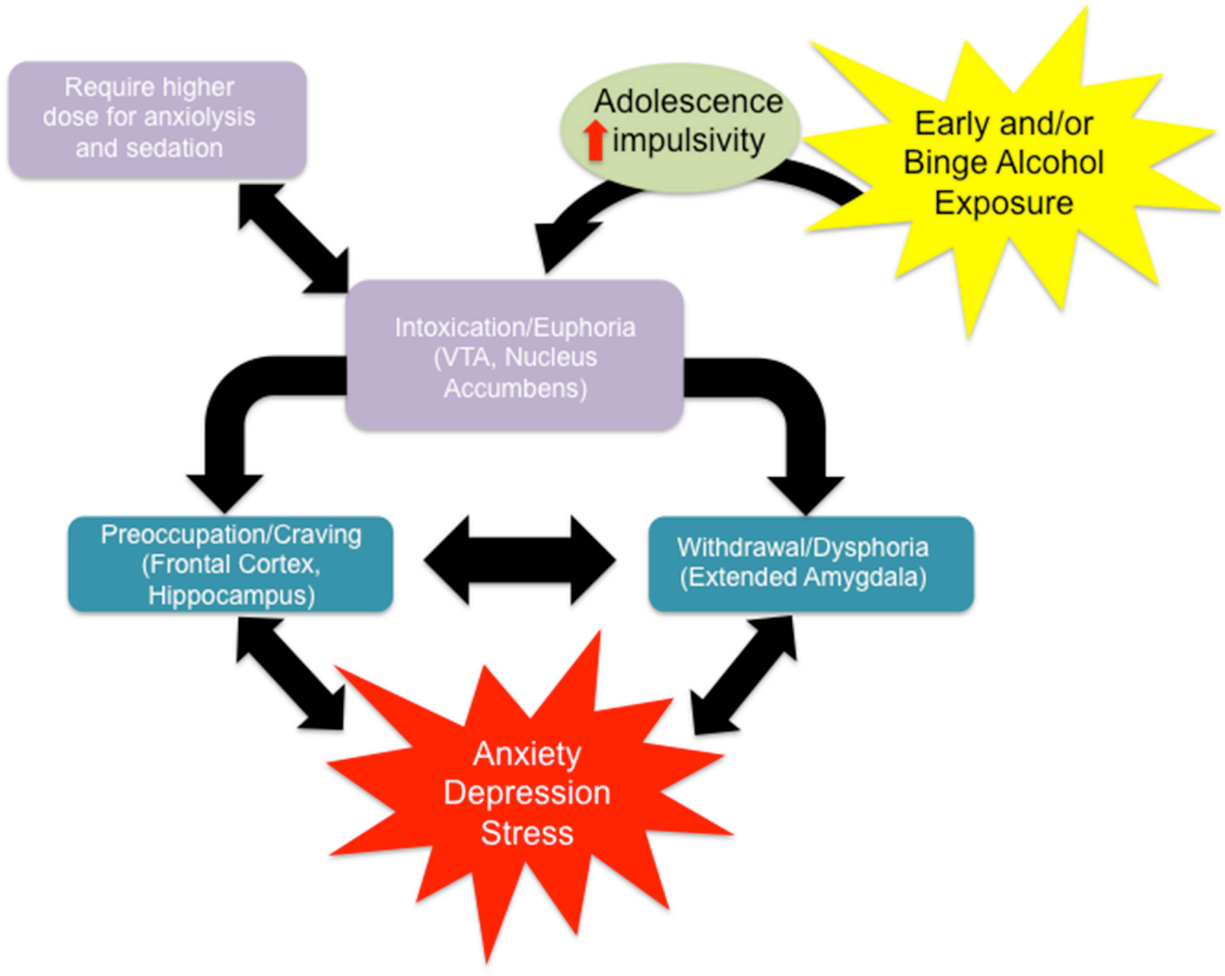
**Alcohol use disorder is characterized by the classical pattern of addiction, namely a cycle from a state of euphoria during alcohol intoxication to that of dysphoria during alcohol withdrawal to one of craving in the absence of acute intoxication**. Craving, with or without withdrawal symptoms, is characterized by preoccupation with obtaining alcohol and anticipation of alcohol use, leading to relapse and a return to the intoxicated state. Certain individual clinical characteristics or psychosocial factors can exacerbate this cycle, driving alcohol use and addiction. For example, impulsivity renders an individual more sensitive to the immediate, rewarding effects of alcohol intake, with minimization of any longer term negative consequences, driving alcohol intake. Adolescence alone is characterized by an increased sensitivity to the rewarding effects of alcohol with a protection from the negative effects relative to aged counterparts, driving alcohol intake for this group. Adolescence is often characterized by impulsivity and together these characteristics facilitate alcohol intake. Also they require higher doses of ethanol to produce anxiolysis and sedation. Withdrawal from alcohol can both induce anxiety or depression symptoms and be worsened by comorbid mood disorders. Anxiety or depression can separately be exacerbated by other stressors, acute or chronic, and has been shown to be increased over the long term by environmental insults during development (early adversity). Stress and/or mood decompensation can also, in the absence of withdrawal dysphoria, directly stimulate craving and relapse, driving the cycle of addiction at either stage.

Recent estimates using DSM-V criteria reveal that the prevalence of AUD was 13.9% in the past year and 29.1% lifetime across all age groups (Grant et al., 2016). However, the same study found that 26.7% of respondents between the ages of 18 and 29 met criteria for AUD in the past year. Approximately 40% of adolescent drug-related visits to the emergency room involve alcohol, and nearly 10% of adolescents report drinking and driving (Grigsby et al., 2016). As drinking in adolescence is widespread, this phenomenon warrants detailed study because early use can lead to dependence and addiction later in life (Donovan, 2004; Nixon and McClain, 2010). Specifically, people who begin drinking before the age of 14 show an increased risk for alcohol abuse and dependence in adulthood (DeWit et al., 2000). Additionally, approximately 10–40% of adolescents across populations engage in frequent binge drinking, where 4 (for women) or 5 (for men) drinks are consumed over 2 h or fewer, and blood ethanol concentrations exceed 80 mg/dL (Miller et al., 2007; López-Caneda et al., 2014). Several preclinical studies also indicate ethanol exposure during early adolescence leads to heightened anxiety and higher alcohol intake in adulthood (Pandey et al., 2015; Van Skike et al., 2015). Adolescent alcohol use can lead to various stages of addiction that can be further exacerbated by other factors such as stress and comorbidity with depression and anxiety (Figure 1; Clark et al., 1997). Scientific investigation of binge alcohol use during the adolescent stage is critical, as it leads to an increased risk for psychiatric disorders including anxiety and alcoholism later in adulthood (Figure 1; Pandey et al., 2015). This review will critically address the notion that the persistent risks and effects of adolescent binge alcohol exposure are due, in part, to the altered structure and organization of synaptic connections possibly due to epigenetic reprogramming and related molecular mechanisms.

## Behavioral effects of adolescent ethanol exposure persist to adulthood

### Behavioral effects of alcohol in adolescents

Adolescents respond to alcohol in quantitatively different ways than adults, given the state of adolescent brain development and the molecular and synaptic correlates of this trajectory. In particular, both clinical and preclinical models support the notion that adolescents are more sensitive to the positive rewarding effects of acute alcohol exposure and less sensitive to negative aspects of alcohol intoxication (Donovan, 2004; Spear and Swartzwelder, 2014). However, adolescents and young adults appear to show greater neural reorganization and degeneration after binge alcohol use than their adult counterparts (Vetreno et al., 2014), suggesting that cellular and molecular mechanisms are involved in the differential responsiveness to ethanol exposure.

Adolescents are less sensitive to the adverse effects of alcohol consumption that often limit heavier drinking patterns in adults (Spear and Varlinskaya, 2005). Adolescents appear to be less sensitive to severe aspects of alcohol withdrawal such as seizures (Chung et al., 2008), which is dependent on the increased metabolic capacity of adolescents (Morris et al., 2010a). In rodents, adolescents show decreased anxiety during the withdrawal period from alcohol compared to adults (Slawecki et al., 2006). Adolescents are less sensitive to the anxiolytic effects of alcohol compared to adult rats (Sakharkar et al., 2012, 2014). In addition, the sedative and motor effects of alcohol exposure are less severe in adolescent animals when compared to adults (Little et al., 1996). Adolescents show an increased responsiveness to the rewarding and positive effects of alcohol consumption (Spear and Varlinskaya, 2005; Spear and Swartzwelder, 2014). Adolescents will self-administer alcohol to the point of tachycardia, unlike adults (Ristuccia and Spear, 2008). Increased alcohol-induced heart rate in young adults is associated with reduced subjective intoxication but increased alcohol-induced mood changes (Conrod et al., 2001), suggesting that the increased self-administration of alcohol by adolescents is connected to its rewarding effects. Alcohol also induces social facilitation more robustly in adolescent animals compared to adults (Varlinskaya and Spear, 2008).

### Persistent effects of adolescent alcohol use on adult behavior

Adolescent binge-like alcohol exposure alters a number of different behaviors in animal models. For example, adolescent alcohol causes deficits in reversal learning, a measure of cognitive flexibility, at adulthood in a rat model. The same study found that adolescent ethanol conferred resistance to the extinction of ethanol self-administration in adulthood (Gass et al., 2014). It is interesting to note that adolescent intermittent ethanol exposure in rats produces anxiety-like behaviors during immediate withdrawal, a phenomenon that persists into adulthood (Sakharkar et al., 2014, 2016; Pandey et al., 2015). However, in adult rats chronic ethanol exposure also produces anxiety-like behaviors, but these behaviors disappear within a few days of last ethanol exposure (Pandey et al., 1999a; Zhang et al., 2007; Aujla et al., 2013).

Adolescent alcohol leads to long-term deficits in novel object recognition in adult animals (Vetreno and Crews, 2015). Multiple studies have shown increased ethanol consumption in adult rodents exposed to adolescent alcohol (Gilpin et al., 2012; Alaux-Cantin et al., 2013; Broadwater and Spear, 2013; Pandey et al., 2015), as well as decreases in alcohol-induced conditioned taste aversion in adulthood (Diaz-Granados and Graham, 2007). Adult mice exposed to binge-like alcohol during adolescence also show resistance to alcohol-induced sedation (Matthews et al., 2008), social impairment (Varlinskaya et al., 2014), and motor impairment (White et al., 2002), but increased levels of alcohol-induced social facilitation (Varlinskaya et al., 2014) and working memory deficits (White et al., 2000). These observations lend themselves to an emerging hypothesis in the field that prolonged and/or repeated binge exposure to alcohol during the critical developmental period of adolescence may cause the persistence of adolescent-like phenotypes, namely the increased responsiveness to positive aspects and decreased responsiveness to negative aspects of intoxication, into adulthood (Spear and Swartzwelder, 2014).

## Adolescent neuromaturation and synaptic organization: Relevance to alcohol exposure

The period of adolescence is particularly important for brain development and involves changes in synaptic structure, gene expression, and neurotransmission in crucial brain circuits responsible for emotion and cognition (Tau and Peterson, 2010). For example, reward seeking behaviors peak in adolescence well before the complete maturation of executive control and regulation networks (Keshavan et al., 2014). This “maturational lag” is underpinned by a complex concert of cellular and molecular changes in the brain involving various neurotransmitters and neural circuits (Keshavan et al., 2014).

**Figure 2:**
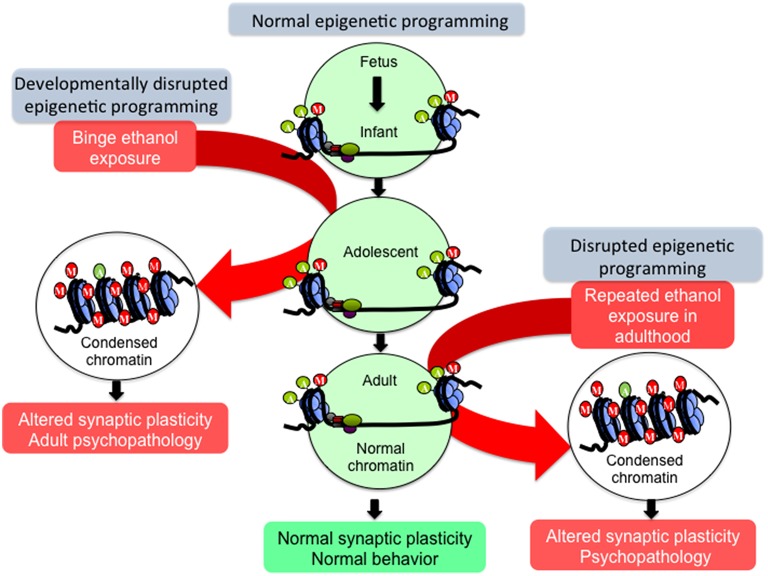
**Hypothetical model of epigenetic reprogramming by alcohol during adolescence**. Adolescence is characterized by widespread changes in the developing brain. These changes are underpinned by molecular processes such as epigenetic programming, which turn specific genetic programs on to mediate cell fate determination, axonal outgrowth, dendrite formation and neuronal maturation. These processes, in turn, affect synaptic plasticity and neurocircuit function to shape normal behaviors. However, developmental perturbation by early life and/or binge alcohol exposure during this critical period can produce persistent effects on chromatin remodeling, synaptic plasticity, and brain function in adulthood, leading to alcohol use disorders (AUDs) and comorbid psychopathology including anxiety and depression. Adult exposure of alcohol can also disturb chromatin remodeling and plasticity, but some of these effects may not be long-lasting.

Adolescents generally exhibit high levels of impulsive behavior (Keshavan et al., 2014), and are additionally highly sensitive to the rewarding effects and less sensitive to the sedative and anxiolytic effects of alcohol (Beck et al., 1993; Sakharkar et al., 2012, 2014; Spear and Swartzwelder, 2014). These characteristics may permit excessive alcohol intake during adolescence, and as mentioned above, adolescent alcohol exposure is a major risk factor for lifetime AUD prevalence (DeWit et al., 2000). Additionally, the earlier the onset of drinking during adolescence, the earlier and more severe the onset of adult AUD (Donovan, 2004; Dawson et al., 2008; Nixon and McClain, 2010). Adolescent alcohol may alter the normal processes of neuromaturation through biological mechanisms including epigenetics, arresting the behavioral development of impulse control and executive function, promoting the maintenance of adolescent-like impulsive use of alcohol into adulthood, intensifying the cycle of addiction, and potentially predisposing an individual to adult psychopathology (Figures 1, 2; Crews et al., 2007; Pascual et al., 2009; Jacobus and Tapert, 2013; Conrod and Nikolaou, 2016).

### Neurocircuitry of alcohol exposure and addiction

The neurocircuitry involved in alcohol abuse and the addiction cycle has recently been comprehensively reviewed (Koob and Volkow, 2010), but we will briefly outline this topic for our purposes. Addiction to alcohol and other drugs is posited to involve three main phases involving distinct brain circuits: (1) binge intoxication, (2) withdrawal/negative affective states, and (3) craving/preoccupation with drug intake (Koob and Volkow, 2010).

Each of these phases involves corresponding neuroadaptive changes in brain circuitry. For example, the ventral tegmental area (VTA), ventral pallidum, and nucleus accumbens (NAc) are involved in the initial binge/intoxication stage of drug and alcohol use. Ethanol directly increases the firing rate of dopaminergic VTA neurons (Brodie et al., 1990), and dopamine and GABA_A_ receptors in the ventral pallidum are likely involved in the reinforcing effects of acute alcohol use (Melendez et al., 2004). Dendritic spine density in the VTA and NAc is critically involved in addiction to alcohol and other drugs of abuse (Spiga et al., 2014a), and chronic alcohol exposure affects synaptic organization in the NAc (Zhou et al., 2007; Spiga et al., 2014b).

The extended amygdala, composed of the central nucleus of the amygdala (CeA), bed nucleus of the stria terminalis (BNST), and a transitional zone of the NAc shell (Alheid, 2003), is proposed to integrate brain stress and reward systems to produce negative affective states during the withdrawal stages of drug and alcohol use and addiction (Koob and Volkow, 2010). Extended amygdala regions receive inputs from the cortex, hippocampus and basolateral amygdala (BLA) and send projection fibers to the ventral pallidum, hypothalamus, and cortical structures (McDonald et al., 1999; Alheid, 2003). Extended amygdala structures display plasticity after exposure to alcohol. For example, repeated ethanol exposure leads to a decrease in dopamine and serotonin release in the NAc 8 h after last ethanol exposure (Weiss et al., 1996) that is again restored with ethanol exposure. Brain stress systems also contribute to neuroadaptations that develop with ethanol exposure to promote a maladapted baseline state of heightened arousal and anxiety-like behaviors in the absence of ethanol. This is, in part, due to ethanol-induced increases in the activity of the corticotrophin releasing factor (CRF) system in the CeA (Funk et al., 2006; Roberto et al., 2010). For example, infusion of a CRF antagonist into the CeA leads to a reduction of ethanol self-administration during withdrawal in ethanol-dependent rats (Funk et al., 2006). CRF activity mediates the behavioral stress response and anxiety-like behaviors during both ethanol withdrawal and protracted abstinence (Funk et al., 2006; Heilig and Koob, 2007).

The third stage of alcohol addiction involves preoccupation with drug taking/intoxication and “craving.” It should be noted that the concept of craving has not been successfully measured clinically and may not correlate with actual relapse (Sinha, 2013). This stage of the cycle is thought to involve neuroadaptation of cortical regions such as the prefrontal (PFC) and orbitofrontal cortex (OFC) as well as limbic structures with cortical connections (Koob and Volkow, 2010). For instance, cue-induced drug reinstatement likely involves reciprocal connections between the BLA and PFC (Schulteis et al., 2000; Everitt and Wolf, 2002), and the preoccupation stage of addiction also involves stress systems such as CRF in the extended amygdala circuitry (Shaham et al., 2003), as centrally-administered CRF antagonists can reduce ethanol self-administration following prolonged abstinence from chronic, high-dose ethanol vapor exposure (Valdez et al., 2002). However, addiction-related changes are not limited to these structures and occur in additional brain regions including the hippocampus (Nestler, 2002; Koob and Volkow, 2010). The changes in neural substrates in response to alcohol and other drugs of abuse demonstrate the remarkable structural and molecular plasticity of neurocircuitry, and many of these changes are susceptible to alterations during adolescence.

### Adolescent neurodevelopment in addiction-related circuitry

Adolescence is marked by widespread changes in the developing brain that include the regions involved in alcohol addiction stated above. Brain white matter increases throughout adolescence as a function of ongoing myelination and gray matter reduction (Gogtay et al., 2004; Shaw et al., 2008). Total cortical volume initially increases during early adolescence before synaptic pruning occurs causing the cortex to decrease in size and reach normal adult proportions (Shaw et al., 2008). Dendritic spines are overproduced during initial neurogenesis and early development and are pruned away in an activity-dependent manner (Changeux and Danchin, 1976). It was once widely accepted that this synaptic pruning was mostly complete by the early adolescent stage of life (Bourne and Harris, 2008), but recent studies have shown that this process continues in cortical and limbic regions well into early adulthood in both humans (Petanjek et al., 2011; Goyal and Raichle, 2013) and animal models (Willing and Juraska, 2015; Johnson et al., 2016).

In addition to early life synaptic remodeling, the refinement of specific brain circuits corresponds to the behavioral changes seen in adolescence. For example, evolutionarily older structures such as the amygdala and hippocampus are among the first brain regions to reach adult-like levels of total volume and synaptic organization, while phylogenetically younger structures such as the PFC and associated cortical areas do not obtain adult-like stability until late adolescence or early adulthood (Gogtay et al., 2004; Shaw et al., 2008). This correlates roughly with an increase in reward-seeking behaviors during adolescence, including drug and alcohol use, as brain areas involved in executive function and complex decision-making have yet to mature while brain areas involved in anxiety and emotionality have reached histological and possibly functional maturity (Casey et al., 2011; Lebel and Beaulieu, 2011).

## Alcohol-induced epigenetic reprogramming and synaptic remodeling

As alcohol exerts potent effects on the brain at the cellular and molecular level, early life alcohol exposure, and especially adolescent binge drinking, may prime the brain for alcohol-related psychopathology later in life via molecular mechanisms such as epigenetic reprogramming (Figure 2). Adolescence involves a concert of epigenetic cascades that prime the brain for functional changes occurring during this period, and some of these epigenetic processes are likely involved in the aforementioned behavioral and synaptic alterations seen during adolescence (Keshavan et al., 2014).

### Epigenetics and the developmental response to ethanol

“Epigenetics” is defined as a pattern of stable changes to the organization and function of a chromosome that result in a specific phenotype but do not change the underlying DNA sequence (Kouzarides, 2007; Berger et al., 2009). These phenotypes include the methylation and acetylation, among other added structural groups, of histone terminal tails around which DNA is wrapped, and also the addition of methyl groups to DNA itself (Kouzarides, 2007). These epigenetic chemical modifications exert effects on transcription, as DNA methylation is most often an inhibitory marker of closed, inaccessible chromatin while histone acetylation is considered a marker of open, active chromatin (Boyes and Bird, 1991; Gräff and Tsai, 2013). In contrast, histone methylation can either activate or inhibit transcription of the underlying DNA sequence depending on the specific histone protein residue that is modified. For instance, histone 3 lysine 4 (H3K4) methylation usually activates transcription while H3K9 methylation is repressive toward transcription (Kouzarides, 2007). Because epigenetic markers and the enzymes that add or remove these markers fluctuate in expression throughout development, it is hypothesized that perturbations during crucial developmental periods may cause widespread genetic dysregulation possibly due to epigenetic reprogramming, affecting normal developmental trajectories and causing the persistence and/or emergence of pathology in adulthood (Figure 2).

Numerous studies have shown that alcohol effects epigenetic pathways, leading to changes in gene expression, synaptic plasticity, dendritic spine morphology, and behavior (Kyzar and Pandey, 2015). Notably, acute alcohol increases dendritic spine density in the CeA and MeA, while withdrawal from alcohol causes a decrease in spinogenesis in the same regions (Pandey et al., 2008a; Moonat et al., 2013; You et al., 2014). These structural changes are correlated with anxiolysis in response to acute alcohol and anxiety-like behavior during withdrawal, suggesting that the morphological changes in the amygdala are reflective of circuits underlying the expression of anxiety-like behavior. Interestingly, acute alcohol potently inhibits histone deacetylases (HDACs), epigenetic enzymes that remove acetyl groups from histone proteins, leading to increased histone acetylation that drives increased gene expression of crucial synaptic plasticity genes such as brain-derived neurotrophic factor (*Bdnf*) and activity-regulated cytoskeletal-associated protein (*Arc*) and subsequently increased dendritic spine density (Pandey et al., 2008a; Kyzar and Pandey, 2015). Again, these increases are associated with acute ethanol-induced anxiolysis (Moonat et al., 2013; Kyzar and Pandey, 2015). The opposite response is seen during alcohol withdrawal, with increased HDAC activity leading to decreased histone acetylation, decreased *Bdnf* and *Arc* expression and decreased dendritic spine density along with increased anxiety-like behaviors (Pandey et al., 2008a; You et al., 2014). Notably, alcohol-preferring (P) rats show an innately increased level of the histone deacetylase isoform 2 (HDAC2) that leads to decreased histone acetylation, decreased *Bdnf* and *Arc* expression and decreased spine density in the CeA and MeA. Infusion of an HDAC2 siRNA into the CeA reverses these molecular effects while normalizing the dendritic spine density, anxiety-like behaviors, and alcohol intake seen in these animals (Moonat et al., 2013). The notion that temporary inhibition of a single epigenetic enzyme can cause proximal effects on neuronal morphology underlines the impact of epigenetic processes on ongoing synaptic plasticity. Given these effects of alcohol exposure in adult animals, multiple studies have investigated both the acute and long-lasting effects of alcohol on brain epigenetic pathways in adolescence that affect dendritic morphology and behavioral phenotypes in adulthood (Sakharkar et al., 2014; Pandey et al., 2015).

### Effects of alcohol on neuroepigenetics in adolescents

Adolescent ethanol exposure causes widespread epigenetic changes, many of which are dose-dependent. Notably, adolescent animals appear to require a higher dose of alcohol than adults to inhibit HDAC activity in the amygdala and achieve anxiolysis acutely, as they are less sensitive to anxiolytic effects of ethanol (Spear and Varlinskaya, 2005; Walker and Ehlers, 2009; Sakharkar et al., 2012, 2014). Adolescent rats exposed to either one or two doses (24 h apart) of 2 g/kg ethanol show inhibition of HDACs and DNA methyltransferases (DNMTs; add methyl groups directly to DNA) in the amygdala and BNST (Sakharkar et al., 2014). Interestingly, two doses of 2 g/kg ethanol causes a decrease in *Dnmt3l* isoform expression in the amygdala but an increase in *Dnmt1* and *Dnmt3a* in the BNST of these same animals (Sakharkar et al., 2014). Binge-like exposure to alcohol during adolescence causes marked anxiety-like behaviors in adolescent rats 24 h after last alcohol exposure during the withdrawal period (Pandey et al., 2015). This anxiety was associated with increased global HDAC activity and increased HDAC2 and HDAC4 isoform expression in the CeA and MeA, leading to decreased levels of activating histone 3 lysine 9 (H3K9) acetylation (Pandey et al., 2015). Some effects of histone modifications, such as increased expression of HDAC2 and deficits in histone H3K9 acetylation in the amygdala, persist in adulthood. As HDAC2 has been shown to regulate spinogenesis and LTP (Guan et al., 2009), it is possible that the AIE-induced increase in HDAC2 expression in the CeA and MeA may be involved in reduced synaptic plasticity and psychopathology in adulthood (Pandey et al., 2015). Adolescent binge-like exposure also increases the activity of histone acetyltransferases (HATs; add acetyl groups to histone proteins) in the PFC and increases both histone acetylation and permissive H3K4 dimethylation at the promoters of the immediate-early and synaptic plasticity-related genes *Cfos, Cdk5*, and *Fosb* (Pascual et al., 2012). These genes have been implicated in the regulation of behavioral and neuronal plasticity (Nestler et al., 1999; Lai and Ip, 2009). Notably, pre-treatment with the HDAC inhibitor sodium butyrate increased the induction of HATs and promoter-specific histone acetylation by binge-like ethanol in the same study (Pascual et al., 2012). Binge-like alcohol exposure also increases total levels of acetylated H3K9, H4K5, and H4K12 while interestingly decreasing levels of H3K4 trimethylation 24 h after last exposure in the medial PFC of adolescent animals (Montesinos et al., 2016).

Epigenetic mechanisms also contribute to the effects of alcohol in clinical adolescent populations. As genetic risk does not fully explain the heritability of AUD and other addiction-related disorders, the influence of epigenetic environmental factors are posited to play a crucial role in AUD pathogenesis and disease progression (Kofink et al., 2013). A recent clinical study identified DNA hypermethylation at the 3′-protein-phosphatase-1G (*PPM1G*) gene as a risk factor for AUD in discordant monozygotic twins (Ruggeri et al., 2015). In an unrelated sample of adolescents, *PPM1G* hypermethylation was associated with impulsive behaviors and escalation of alcohol intake (Ruggeri et al., 2015). PPM1G interestingly dephosphorylates protein members of the microRNA processing pathway, another epigenetic mechanism responsible for gene regulation and implicated in synaptic organization (Petri et al., 2007; Smalheiser, 2008).

### Persistent effects of adolescent alcohol use on epigenetic pathways in adulthood

**Figure 3:**
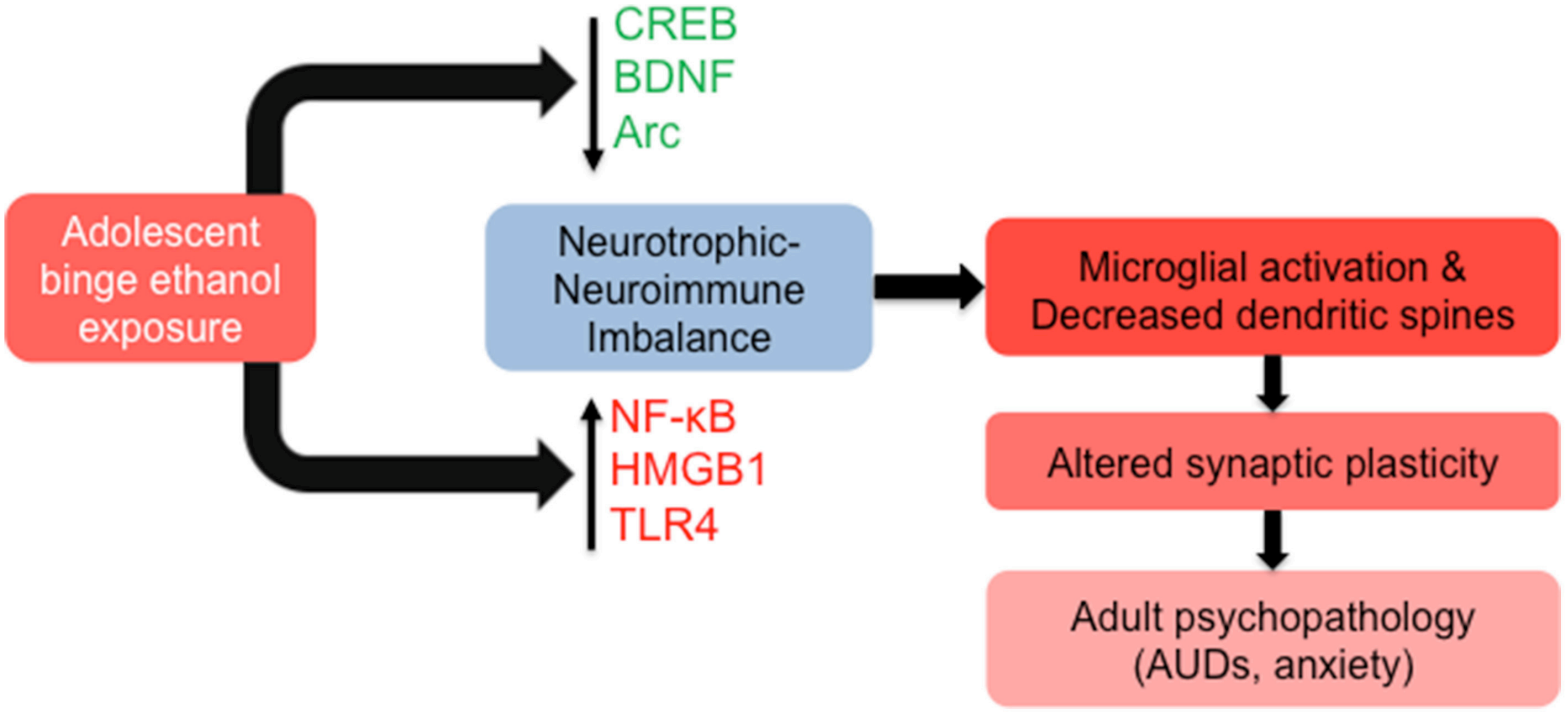
**Molecular and cellular signaling mechanisms in the brain leading to altered synaptic plasticity in adulthood after adolescent alcohol exposure**. Adolescent binge alcohol exposure exerts effects in the brain long after alcohol has left the system. For example, adolescent alcohol causes a long-lasting decrease in key synaptic plasticity-related genes including cyclic AMP response element binding protein (CREB) pathway (CREB binding protein), brain-derived neurotrophic factor (BDNF), and activity-regulated cytoskeleton-associated protein (Arc). At the same time, adolescent binge alcohol exposure increases neuroimmune mediators including nuclear factor kappa-light-chain-enhancer of activated B cells (NF-κB), high mobility group box 1 (HMGB1) and toll-like receptor 4 (TLR4). The increase in neuroimmune factors and decrease in neurotrophic factors may be responsible for molecular imbalance in the adult brain that is mediated by increased microglial activation and decreased synaptic plasticity, leading to an increased risk for alcohol use disorders (AUDs) and related psychopathology such as anxiety.

Many of the long-lasting effects of adolescent alcohol in the brain are likely to be mediated by altered epigenetic programming during crucial stages of development (Figure 2). In adulthood, this manifests as altered epigenetic architecture, specifically around genes that are important for synaptic plasticity such as *Bdnf* and *Arc*. Adolescent intermittent ethanol (AIE) exposure leads to long-lasting increases in global HDAC activity, as well as specific increases in HDAC2 protein and mRNA, in the amygdala at adulthood (Pandey et al., 2015). This is associated with decreased H3K9 acetylation globally and at the promoter regions of *Bdnf* and *Arc* and markedly reduced dendritic spines in the CeA and MeA, but not the BLA (Pandey et al., 2015). Notably, the increased alcohol preference and anxiety-like behaviors seen in AIE-treated animals in adulthood are effectively reversed by treatment with the pan-HDAC inhibitor trichostatin A (TSA) (Pandey et al., 2015), further connecting epigenetic processes to brain morphology and behavior. The same alcohol exposure paradigm caused increased HDAC activity and decreased CREB binding protein (CBP) and histone H3K9 acetylation in the CA1, CA2, and CA3 regions of the hippocampus in adulthood. This was associated with a decrease in BDNF protein and H3 acetylation at the *Bdnf* exon IV promoter that were again reversed by TSA treatment (Sakharkar et al., 2016). Notably, HDAC2 overexpression in the hippocampus is directly linked to increased dendritic spine density, while decreased HDAC2 expression leads to decreased spines and synaptic plasticity as measured by LTP (Guan et al., 2009). Recent studies have linked AIE with increased hippocampal spine density, specifically increased immature dendritic spines, in adulthood (Risher et al., 2015), again highlighting the interrelatedness of epigenetics and synaptic function. AIE exposure additionally increased levels of acetylated H4K5 in the adult medial PFC, with no alterations seen in acetylated H3K9, acetylated H4K12, or trimethylated H3K4 residues, an effect that was abolished in toll-like receptor 4 (*Tlr4*) knockout animals (Montesinos et al., 2016). Therefore, epigenetic alterations induced by adolescent alcohol exposure may be brain region- and residue-specific. These results also suggest that the neuroimmune and epigenetic pathways are interrelated with regards to the lasting effects of adolescent binge-like ethanol, as TLR4 is altered by AIE and other methods of alcohol exposure (Figure 3).

The long-lasting alterations induced in the brain include decreases in BDNF expression in the amygdala and hippocampus of adults that were exposed to adolescent alcohol, and this decrease is regulated by epigenetic mechanisms (Pandey et al., 2015; Sakharkar et al., 2016). The decrease in BDNF and Arc is correlated with decreased dendritic spines in the CeA and MeA (Pandey et al., 2015). In addition to the regulation of *Bdnf* expression by histone acetylation, BDNF is also regulated by a number of different small non-coding RNAs, including microRNAs, that fine-tune cellular levels of this crucial neurotrophin (Numakawa et al., 2011). MicroRNAs are known to be altered in the brain of human alcoholics, as well as in several brain regions of preclinical models, and are likely involved in synaptic remodeling after alcohol exposure (Nunez and Mayfield, 2012; Pandey, 2016). The cyclic AMP response element binding protein (CREB) appears to regulate gene expression via a regulatory loop, with CREB/pCREB/CBP/p300 affecting epigenetic remodeling via histone acetylation, which in turn regulates the expression of crucial pathway genes such as *Cbp*. We recently observed that acute ethanol-induced anxiolysis appears to be regulated by decreased expression of microRNA-494, and increased expression of its target genes *Cbp* and *p300* in the amygdala of adult rats (Teppen et al., 2015). As CREB functions to regulate synaptic plasticity on its own and through the expression of CREB-target genes including *Bdnf* and *Arc*, we hypothesize that the CREB pathway may integrate signals from neuroimmune, neurotrophic, and epigenetic mechanisms to exert persistent effects on dendritic organization and remodeling in response to adolescent binge alcohol exposure (Figure 3; see below).

In addition to lasting effects on histone acetylation, histone methylation mechanisms have recently been examined in the adult amygdala after AIE exposure. mRNA of the neuron-specific splice variant of lysine demethylase 1 (Lsd1), termed *Lsd1* + *8a* for the inclusion of the mini-exon 8a, is decreased in the CeA and MeA of adult animals following binge-like alcohol exposure in adolescence (Kyzar et al., 2016). This leads to increased repressive H3K9 dimethylation in the CeA and MeA, but unchanged activating H3K4 dimethylation in the amygdala, which correlates with increased anxiety-like behaviors and possibly exerts effects on synaptic plasticity and morphology (Kyzar et al., 2016). Notably, the neuron-specific *Lsd1* + *8a* regulates neurite outgrowth during neuronal development, and recent *in vitro* studies suggest that this splice variant primarily works to demethylate H3K9 residues and not H3K4 (Zibetti et al., 2010; Laurent et al., 2015). The involvement of histone methylation mechanisms in the long-lasting effects of adolescent alcohol exposure highlights the need for investigation of additional epigenetic modifications after alcohol exposure, as some may be more sensitive to the perturbation of normal epigenetic and developmental trajectories and thus better targets for therapeutic intervention. For example, recent work shows that escalating alcohol consumption following ethanol vapor exposure causes progressive increases in DNA methylation at crucial synaptic plasticity genes such as synaptotagmin 2 (*Syt2*) in the medial PFC that is reversed by local infusion of a DNMT inhibitor (Barbier et al., 2015).

### Alcohol-induced changes in the adolescent brain: Neurogenesis

Adult human alcoholic patients show characteristic structural brain abnormalities including presumed neurodegeneration in response to heavy and prolonged alcohol use (Crews and Nixon, 2009), and some of these effects may be regulated by epigenetic mechanisms. Patients with adolescent-onset AUD demonstrate marked reductions in total hippocampal and PFC volume (De Bellis et al., 2000; Medina et al., 2008), with hippocampal alterations uncharacteristic of adult-onset AUD. Studies in animal models have shown that a 4-day alcohol binge significantly reduces markers of neurogenesis in the hippocampus, including doublecortin (DCX) staining (a marker of immature neurons) and bromodeoxyuridine (BrdU) staining of DNA replication events (Morris et al., 2010b). It is possible that decreased adolescent neurogenesis may lead to the reductions in hippocampal volume in early-onset AUD patients (Donovan, 2004). Adolescent binge drinking damages white matter tracts in humans, and this effect correlates with estimated blood alcohol concentrations (Jacobus et al., 2009). The disruption in white matter integrity is also present in adolescents who show subclinical levels of binge drinking (i.e., not qualifying for AUD) (Jacobus et al., 2009). Alterations in hippocampal and PFC-related circuitry are manifested behaviorally by cognitive impairments in binge-drinking adolescents. For example, binge drinkers demonstrate decreased performance on simple verbal memory tasks while showing increased utilization of higher order cortical areas, possibly to compensate for decreased hippocampal and medial temporal lobe utilization (Tapert et al., 2004; Schweinsburg et al., 2010). Adolescent ethanol also exerts negative effects on learning and memory in animal models (Schulteis et al., 2008), suggesting that the behavioral manifestations of altered brain structure may be useful biomarkers for early intervention.

### Persistent effects of adolescent alcohol use on adult neurogenesis

The persistent behavioral effects of adolescent alcohol are accompanied by marked effects on synaptic plasticity and neurotransmission. Adolescents show enhanced long-term potentiation (LTP), or the ability of synaptic activity to increase downstream signaling effects and ion flux during subsequent activation, at baseline compared to adult animals (Johnston, 1995), and consequently adolescents show a greater decrease in LTP after acute ethanol exposure than adult animals (Swartzwelder et al., 1995). Interestingly, animals exposed to binge-like ethanol (5 g/kg ethanol via intragastric gavage [i.g.] on a 2 days on, 1 day off, 2 days on, 2 days off schedule) during adolescence display enhanced LTP in the CA1 region of the hippocampus at adulthood (Risher et al., 2015). This is associated with increased dendritic spine density, but a predominance of immature spines and decreased synaptic connections and synaptic scaffolding proteins postsynaptic density 95 (PSD-95) and synapse-associated protein 102 (SAP-102) (Risher et al., 2015). Additional studies have shown a decrease in dendritic spine density in the CeA and MeA, which are primarily GABAergic in nature, in adult animals exposed to AIE via i.p. injections (Pandey et al., 2015). Notably, the BLA, which is primarily glutamatergic, did not show gross alterations in spines at adulthood, suggesting that a cell-type specific mechanism may target specific cells during high-dose adolescent alcohol consumption.

Neurogenesis is another phenomenon of brain plasticity that continues to occur in the adult brain in discrete locations including the subventricular zone and the granule cell layer of the hippocampal dentate gyrus (Eriksson et al., 1998). Numerous studies have reported a decrease in markers of neurogenesis, including DCX staining and Ki-67 staining (a marker of neural progenitor cell proliferation), in the hippocampus of adult animals exposed to intermittent binge-like alcohol by both i.p. and i.g. routes of administration in adolescence (Broadwater et al., 2014; Vetreno and Crews, 2015; Sakharkar et al., 2016), an effect that does not persist in adult rats exposed to similar amounts of ethanol (Broadwater et al., 2014). The decrease in neurogenesis in these animals may contribute to some of the long-lasting behavioral abnormalities, specifically those related to memory, cognition and anxiety, observed in AIE adult animals. Of particular interest is the increased nuclear HDAC activity and decreased markers of hippocampal neurogenesis (DCX and Ki-67) seen in adult rats exposed to AIE (Sakharkar et al., 2016). Similar to the epigenetic changes seen in these animals in the amygdala (Pandey et al., 2015), TSA administration reverses deficits in histone acetylation of the *Bdnf* gene and rescues the decrease in neurogenesis markers in the hippocampus (Sakharkar et al., 2016). These studies again emphasize the emerging role of epigenetics in regulating synaptic plasticity, and epigenetic drugs such as HDAC inhibitors may hold promise as therapeutic interventions for AUDs and other psychiatric disorders in adulthood (Pandey et al., 2008a, 2015; Kofink et al., 2013; You et al., 2014; Kyzar and Pandey, 2015).

## Acute and lasting effects of adolescent alcohol on neurotransmission and neuroimmunity

### Effects of alcohol on neurotransmission in adolescents

The behavioral effects that differentiate the adolescent and adult response to ethanol are underpinned by cellular and molecular changes in the brain. For example, the decreased sensitivity to the negative aspects of alcohol intoxication may be a function of decreased facilitation of GABAergic inhibitory postsynaptic currents (iPSCs) in the hippocampus of adolescents vs. adult animals (Li et al., 2003, 2006). As GABA receptors play a crucial role in synaptic reorganization during neurogenesis and after exposure to stressful stimuli (Inoue et al., 2013), reduced alcohol-induced potentiation of iPSCs may contribute to alterations in dendritic dynamics following alcohol exposure.

The increased sensitivity to the positive effects of alcohol may be mediated by increased alcohol-induced dopamine release in the NAc in adolescents (Pascual et al., 2009). Following alcohol exposure, adolescent animals display increased baseline dopamine release in the NAc and decreased dopamine receptor D2 expression. Conversely, alcohol induces a decreased accumbal dopamine response in these animals (Pascual et al., 2009; Philpot et al., 2009). The increased basal dopamine, decreased alcohol-induced dopamine, and decreased D2 expression is hypothesized to contribute to a state of reward deficit, correlating with high levels of risk-taking behavior typically seen during adolescence (Koob et al., 2014). Normally, dopamine release in the NAc peaks during adolescence before falling to mature levels (Keshavan et al., 2014). Adolescent alcohol exposure may alter the normal developmental pattern of the VTA, NAc, and related systems via synaptic remodeling, which plays a crucial role in the switch from casual drug-taking to dependence for alcohol and other drugs of abuse (Spiga et al., 2014b).

Notably, dopamine interacts with glutamate in the NAc in an age-dependent manner. Dopamine appears to decrease the magnitude of NMDA-receptor mediated excitatory postsynaptic currents (ePSCs) in adolescents, but facilitate ePSCs in adult NAc slices in a mechanism dependent on presynaptic D1 receptors (Huppé-Gourgues and O'Donnell, 2012; Zhang et al., 2014). The glutamatergic system is heavily implicated in the synaptic pruning that occurs during development, contributing to the fluctuations in cortical volume seen during adolescence (Johnston, 1995; Crews et al., 2007). Adolescents show increased inhibition of NMDA-mediated synaptic activation compared to adult animals in the hippocampus (Swartzwelder et al., 1995). The NMDA receptor consists of multiple subunits that dimerize to produce mature receptors, with NR2A, NR2B, or another subunit coupling with the requisite subunit NR1. Interestingly, NR2B predominates in the developing brain and has a greater affinity for downstream calcium-related signaling mechanisms than NR2A, which increases in expression across development to become the most prominent subunit in the adult brain (Williams et al., 1993; Tovar and Westbrook, 1999). This developmental switch also plays a role in synaptic maturation and elimination (Barria and Malinow, 2005; Gambrill and Barria, 2011). Adolescent alcohol exposure changes the proportion of NR2A and NR2B receptors in multiple brain regions including the hippocampus, NAc, and cortical areas across multiple routes of administration including i.p. injection, vapor inhalation, and *ad libitum* consumption (Hargreaves et al., 2009; Pascual et al., 2009; Pian et al., 2010).

### Neurotransmission effects caused by adolescent alcohol in adulthood

Tonic inhibition by GABA, mostly mediated by extrasynaptic receptors, is also reduced in normal adolescent hippocampal slices when compared to adults, but is more sensitive to potentiation by ethanol (Fleming et al., 2007). Adult hippocampal slices taken from rats exposed to adolescent alcohol (5 g/kg ethanol via intragastric gavage [i.g.] on a 2 days on and 2 days off schedule) show the same pattern of baseline reduction and increased ethanol-induced potentiation of GABA-mediated tonic inhibition when compared to control animals (Fleming et al., 2012, 2013). However, the same exposure paradigm decreased adult hippocampal protein levels of the GABA_A_ α4 receptor and the extrasynaptic GABA_A_ δ receptor (Centanni et al., 2014). The GABA_A_ δ receptor-mediated tonic inhibitory current is furthermore reduced in the adult prelimbic cortex of animals exposed to intermittent alcohol in adolescence despite the observation that expression of this receptor does not change (Centanni et al., 2016).

Adolescent alcohol exposure additionally exerts lasting effects on dopaminergic signaling. Adult animals exposed to moderate doses of ethanol in adolescence show increased stimulus-evoked mesolimbic dopamine release as measured by fast-scan cyclic voltammetry that may impact reward-associated decision making (Spoelder et al., 2015). AIE-exposed (via intermittent i.g. exposure) adult animals show decreased ethanol-evoked dopamine release in the NAc at adulthood (Shnitko et al., 2016). Additionally, adult rats show a stark decrease in markers of cholinergic (choline acetyltransferase; ChAT) neurons in the basal forebrain and dopaminergic (tyrosine hydroxylase; TH) neurons in the prelimbic cortex in adulthood after AIE via i.g. route of adminstration (Boutros et al., 2014; Vetreno et al., 2014). Notably, the decrease in ChAT staining in the basal forebrain is only persistent after adolescent binge exposure and not after exposure to the same dose and timing of alcohol exposure in early adulthood (Vetreno et al., 2014).

### Effects of alcohol on neuroimmune activation in adolescents

Mediators of the immune response additionally play a role in the cellular response to alcohol, especially during adolescence (Crews and Vetreno, 2016). Younger age of alcohol drinking onset correlates with increased expression of immune-related genes including receptor for the advanced glycation end product (*RAGE*), high-mobility group box 1 (*HMGB1*), and the HMGB1 receptor *TLR4* in the postmortem brains of human alcoholics (Vetreno et al., 2013). Animal models show that binge ethanol exposure (5 g/kg i.g. ethanol on a 2 days on-2 days off schedule) increases *TLR4* and *HMGB1* in the PFC of adolescent rats (Vetreno and Crews, 2012). An additional study demonstrated that binge-like alcohol increases *TLR2* and *TLR4* in the PFC along with the inflammatory cytokines tumor necrosis factor alpha (*TNF*α) and interleukin 1 beta (*IL-1*β) (Pascual et al., 2014). Both the HMGB1-TLR4 and IL-1β signaling cascades mediate increased flux through synaptic NMDA receptors that increases the likelihood for excitotoxicity (Viviani et al., 2003; Balosso et al., 2014). Chemokine (C-C motif) ligand 2 (*Ccl2*) gene expression is increased in the cortex of adolescent rats during withdrawal to a greater extent than adults (Harper et al., 2015), but an earlier study in mice found that *Ccl2* mRNA was increased in the adult but not adolescent cortex, hippocampus, and cerebellum (Kane et al., 2014). Interestingly, many of the epigenetic alterations seen in the brain of mice exposed to binge-like levels of alcohol were abolished in *Tlr4* knockout mice, suggesting that the epigenetic and neuroimmune systems interact during adolescent alcohol exposure (Montesinos et al., 2016).

### Persistent effects of adolescent alcohol use on neuroinflammation in adulthood

Alcohol potently activates neuroimmune pathways in both the developing and adult brain, but some of these effects continue to persist after adolescent alcohol exposure into adulthood. The upregulation of HMGB1 and TLR4 by adolescent intermittent alcohol exposure (i.g) persists into adulthood in the cortex (Vetreno and Crews, 2012). Notably, mice lacking the *Tlr4* gene appear to be additionally resistant to the long-term lasting effects of AIE exposure on epigenetic remodeling and cognitive functioning (Montesinos et al., 2016). A 4-day alcohol binge during adolescence is sufficient to cause activation of microglial cells that persists for at least 30 days (McClain et al., 2011), which is important due to the well-studied effects of microglia on synaptic pruning and may play a role in ethanol-induced structural remodeling (Yang et al., 2014). Recent work suggests that lifetime ethanol exposure positively correlates with brain neuroimmune markers such as HMGB1 and TLR4 (Vetreno et al., 2013), and adolescent binge alcohol use may predispose an individual to an addictive cycle of alcohol abuse resulting in steadily increasing immune activation, likely mediated at least in part through epigenetic and chromatin regulation, and subsequent synaptic remodeling (Figure 3).

## Convergence of molecular and synaptic targets after adolescent alcohol exposure

Adolescent alcohol exposure causes specific long-term changes to neurocircuitry across the domains of immune function, neurogenesis, and epigenetic programming (Vetreno et al., 2013; Pandey et al., 2015; Vetreno and Crews, 2015; Sakharkar et al., 2016). Although the studies described above have generally focused on biological parameters relevant to one domain, in actuality these systems likely act in concert to exert lasting effects on synaptic function and behavior. The p65 isoform of the nuclear factor kappa-light-chain-enhancer of activated B cells (NF-κB) is increased in the adult medial PFC of mice exposed to binge-like ethanol (8 total i.p. injections of 3 g/kg ethanol on a 2 days on-2 days off schedule) in adolescence, an effect that is not present in *Tlr4* receptor knockout mice (Montesinos et al., 2016). These changes coincide with epigenetic alterations in the medial PFC that are not present in *Tlr4* knockout mice (Montesinos et al., 2016). Notably, NF-κB and TLR4 are known to interact with two genes that are crucially involved in the actions of alcohol: *CREB* and *BDNF* (Marini et al., 2004; Kaltschmidt et al., 2006).

### CREB as a master regulator of alcohol action and synaptic plasicity

CREB is a gene transcription factor that binds to cAMP response elements (CRE) on DNA after phosphorylation and usually increases the transcription of downstream genes such as *Bdnf* and *Arc*. The molecular actions of CREB in the development of addiction have been extensively studied, providing evidence of a common molecular mechanism for addictive behaviors (Pandey, 2004; Nestler, 2005). As NF-κB is known to interact with CREB and influence synaptic plasticity (Marini et al., 2004; Kaltschmidt et al., 2006), these two transcription factors may act in convergence to remodel neural circuits and synapses after high-dose adolescent alcohol exposure. As described above, CREB also interacts with CBP and p300, both of which act as HATs, to remodel chromatin in response to alcohol exposure (Pandey et al., 2008a; Teppen et al., 2015), possibly integrating neuroimmune, epigenetic, and neurotrophic pathways to influence synaptic plasticity and behavior (Figure 3).

CREB functioning within the amygdala plays an integral role in the development and maintenance of AUD. It has been shown that alcohol-preferring (P) rats display innately heightened anxiety levels and excessive alcohol drinking behaviors, as well as lower levels of CREB and the functionally active phosphorylated form of CREB (pCREB) within the CeA and MeA when compared to non-preferring (NP) rats (Pandey et al., 1999b, 2005). Additionally, a single acute alcohol exposure (1 g/kg; i.p.) produces anxiolysis and activation of amygdalar CREB leading to increased expression of BDNF and dendritic spines in both P rats and an unselected stock of rats, but not in NP rats (Pandey et al., 2005, 2008b; Moonat et al., 2011). Furthermore, innately lower levels of CREB and pCREB have been found in the NAc shell in a genetic strain of alcohol preferring mice (C57BL/6J) as compared to non-preferring DBA/2J mice (Belknap et al., 1993; Misra and Pandey, 2003). Recently, we observed that decreased DNA demethylation mechanisms are associated with decreased *Bdnf* expression in the NAc shell while also promoting alcohol drinking behavior (Gavin et al., 2016). Several studies demonstrated that CREB phosphorylation increases during acute alcohol exposure, normalizes in response to chronic alcohol treatment, and decreases in amygdaloid structures during alcohol withdrawal, suggesting that CREB serves as a molecular switch in the CeA to regulate anxiety and alcohol drinking behaviors (Pandey et al., 2003, 2005, 2008b; Moonat et al., 2011).

### CREB target genes and alcohol

CREB-regulated pathways in the amygdala regulate alcohol-related behaviors (Pandey, 2004; Teppen et al., 2015). An important target of CREB, the neurotrophic factor BDNF, is activated by CREB plays important roles in neuronal development, neurogenesis, synaptic plasticity, and regulation of dendritic morphology (Poo, 2001). Interestingly, BDNF is regulated by its own signaling pathway via activation of tropomyosin receptor kinase B (TrkB) receptors and mitogen activated protein (MAP) kinases that induce CREB phosphorylation (Finkbeiner et al., 1997; Poo, 2001; Minichiello et al., 2002).

Ethanol increases *Bdnf* expression, and BDNF signaling pathways in specific brain regions play a profound role in regulating alcohol drinking and anxiety-like behaviors (McGough et al., 2004; Pandey et al., 2006; Prakash et al., 2008; You et al., 2014; Warnault et al., 2016). For example, decreased *Bdnf* expression in hippocampal and amygdaloid brain regions results in increased ethanol consumption, while increased *Bdnf* expression attenuates ethanol intake (McGough et al., 2004; Pandey et al., 2006). It has been shown recently that decreased function of BDNF in the medial prefrontal cortex due to a BDNF valine 68 to methionine (Val68Met, analogous to the human Val66Met) polymorphism increases alcohol-drinking behaviors in mice (Pandey, 2016; Warnault et al., 2016). BDNF expression and phosphorylation of members of its signaling cascade, namely MAP kinases and CREB, are increased in CeA and MeA by acute ethanol, which is normalized after chronic treatment and decreased significantly during ethanol withdrawal in rats (Pandey et al., 2008b). Moreover, intra-CeA infusion of BDNF attenuated anxiety-like behaviors that developed during ethanol withdrawal in rats (Pandey et al., 2008b). Further studies suggest that BDNF acts through Arc, which ultimately regulates spinogenesis as *Arc* expression is decreased in the CeA during ethanol withdrawal and normalized by BDNF infusion (Pandey et al., 2008b). It has been shown that *Arc* antisense oligodeoxynucleotide (ODN) infusion into CeA significantly decreases *Arc* expression, dendritic spines, and promotes anxiety-like and alcohol drinking behaviors in rats (Pandey et al., 2008b). The direct role of BDNF in various amygdaloid nuclei in anxiety-like and alcohol drinking behaviors has been well established (Pandey et al., 2006). Infusing BDNF antisense ODN directly into the CeA or MeA, but not in the BLA, is associated with increased ethanol consumption and provokes anxiety-like behaviors in rats. However, these behavioral changes were prevented following co-infusion of exogenous BDNF with the BDNF antisense ODN (Pandey et al., 2006). It is important to point out that BDNF antisense ODN infusion in each of the amygdaloid nuclei significantly decreased BDNF expression and phosphorylation of CREB and MAP kinases that were reversed by co-infusion with exogenous BDNF (Pandey et al., 2006). Baseline BDNF mRNA and protein levels are lower in the CeA, MeA, and bed nucleus of stria terminalis, but not BLA and NAc shell or core, of P vs. NP rats (Prakash et al., 2008). Moreover, acute ethanol exposure increased BDNF and Arc expression and dendritic spines in the CeA and MeA, but not BLA of P rats, leading to anxiolytic-like effects (Moonat et al., 2011). Several other studies have found that deficits in BDNF in PFC, hippocampal, and striatal brain regions are also involved in regulating alcohol consumption in animals (McGough et al., 2004; Logrip et al., 2015; Pandey, 2016; Warnault et al., 2016). Exogenous BDNF exposure appears to play a protective role in ethanol-induced cytotoxic damage in cultured neuronal cells (Sakai et al., 2005). Human studies show that decreased serum BDNF levels in alcoholics are associated with alcohol withdrawal symptoms (Joe et al., 2007; Huang et al., 2011), and *BDNF* gene polymorphisms have been linked to increased susceptibility to alcohol abuse (Uhl et al., 2001; Matsushita et al., 2004; Warnault et al., 2016). As discussed above AIE also produces long-lasting reductions in the expression of BDNF in the amygdaloid and hippocampal brain regions of rats during adulthood and these changes are correlated with phenotypes of anxiety and alcohol intake (Pandey et al., 2015; Sakharkar et al., 2016). Taken together, these results provide evidence of homeostatic mechanisms utilized by the BDNF signaling pathway in various key brain circuits in the regulation of alcohol addiction, and these mechanisms are critical in the development of alcohol dependence and promoting drinking (Pandey, 2016).

## Future directions and conclusion

Scientific inquiry into the adolescent alcohol exposure and its lasting effects has increased greatly in recent years. As human populations are often confounded with variable onset of drinking, polydrug use, and other factors, animal models have been utilized in the vast majority of mechanistic studies presented here. Additionally, these animal models have often used only male rats. As the influence of sex differences in alcohol response has recently been critically reviewed (Becker and Koob, 2016), it is crucial to use both male and female cohorts to examine the acute and lasting effects of adolescent alcohol abuse. Future studies should attempt to translate the most robust and reproducible preclinical epigenetic findings into human populations, possibly establishing novel treatments for AUDs.

As many of the studies cited herein have used different modes and schedules of alcohol exposure, it is important to note that some effects of adolescent exposure may be specific to routes of administration and exposure paradigms. However, a subset of data advocates that certain long-lasting effects of adolescent alcohol exposure (biochemical and behavioral) occur across treatment paradigms including i.p. injection, i.g. gavage, vapor exposure, and voluntary consumption, and are therefore likely the result of ethanol exposure itself (Hargreaves et al., 2009; Pascual et al., 2009; Pian et al., 2010; Gilpin et al., 2012; Boutros et al., 2014; Broadwater et al., 2014; Pandey et al., 2015; Vetreno and Crews, 2015; Sakharkar et al., 2016).

Taken together, early onset of alcohol use and repeated binge-like exposure during the critical developmental period of adolescence greatly increase the risk for later AUD diagnosis. Animal models have greatly increased our understanding of the biological mechanisms underlying synaptic plasticity associated with this risk. Adolescent alcohol increases neuroimmune signaling in the brain and persistently inhibits markers of neurogenesis in the hippocampus. Additionally, binge-like exposure to alcohol alters crucial epigenetic and neurotrophic factors signaling in multiple brain regions involved in addiction. These molecular factors exert robust effects on neuronal morphology and synaptic plasticity. The altered dendritic spine density and synaptic signaling observed alter adolescent ethanol exposure contribute to alterations in neurocircuitry and behavior, and targeting these cellular mechanisms may lead to new treatments for AUDs and other related psychopathology (Kyzar and Pandey, 2015; Pandey et al., 2015; Kyzar et al., 2016).

## Author contributions

The concept and idea of the review article was conceived by SP and then discussed with all authors. All authors (EK, CF, TT, and SP) contributed in the review of literature and writing of the article. The content of figures was conceived by SP and then prepared and edited by all authors.

### Conflict of interest statement

SP reports that a US patent application entitled “Histone acetyltransferase activators and histone deacetylase inhibitors in the treatment of alcoholism” (serial number 60/848237 filed on September 29th, 2006) is currently pending. The other authors declare that the research was conducted in the absence of any commercial or financial relationships that could be construed as a potential conflict of interest.
